# Epithelial-mesenchymal plasticity is a decisive feature for the metastatic outgrowth of disseminated WAP-T mouse mammary carcinoma cells

**DOI:** 10.1186/s12885-015-1165-5

**Published:** 2015-03-26

**Authors:** Claudia Maenz, Eva Lenfert, Klaus Pantel, Udo Schumacher, Wolfgang Deppert, Florian Wegwitz

**Affiliations:** 1 Institute for Tumor Biology, University Medical Center Hamburg-Eppendorf (UKE), D-20246 Hamburg, Germany; 2 Department of Tumor Virology, Heinrich-Pette-Institute, Leibniz Institute for Experimental Virology, D-20251 Hamburg, Germany; 3 Institute of Anatomy and Experimental Morphology, University Medical Center Hamburg-Eppendorf (UKE), D-20246 Hamburg, Germany; 4 Department of Translational Cancer Research, University Medical Center Göttingen, D-37075 Göttingen, Germany

**Keywords:** Breast cancer, Mammary carcinoma, Metastasis, Tumor cell dissemination, WAP-T mouse, Epithelial-mesenchymal transition EMT, Epithelial-mesenchymal plasticity EMP, Disseminated tumor cell DTC, Circulating tumor cell CTC

## Abstract

**Background:**

Experimental analysis of the metastatic cascade requires suitable model systems which allow tracing of disseminated tumor cells and the identification of factors leading to metastatic outgrowth in distant organs. Such models, especially models using immune-competent mice, are rather scarce. We here analyze tumor cell dissemination and metastasis in an immune-competent transplantable mouse mammary tumor model, based on the SV40 transgenic WAP-T mouse mammary carcinoma model.

**Methods:**

We orthotopically transplanted into immune-competent WAP-T mice two tumor cell lines (H8N8, moderately metastatic, and G-2, non-metastatic), developed from primary WAP-T tumors. G-2 and H8N8 cells exhibit stem cell characteristics, form homeostatic, heterotypic tumor cell systems *in vitro*, and closely mimic endogenous primary tumors after orthotopic transplantation into syngeneic, immune-competent WAP-T mice. Tumor cell transgene-specific PCR allows monitoring of tumor cell dissemination into distinct organs, and immunohistochemistry for SV40 T-antigen tracing of single disseminated tumor cells (DTC).

**Results:**

While only H8N8 cell-derived tumors developed metastases, tumors induced with both cell lines disseminated into a variety of organs with similar efficiency and similar organ distribution. H8N8 metastases arose only in lungs, indicating that organ-specific metastatic outgrowth depends on the ability of DTC to re-establish a tumor cell system rather than on invasion *per se*. Resection of small tumors (0.5 cm^3^) prevented metastasis of H8N8-derived tumors, most likely due to the rather short half-life of DTC, and thus to shorter exposure of the mice to DTC. In experimental metastasis by tail vein injection, G-2 and H8N8 cells both were able to form lung metastases with similar efficiency. However, after injection of sorted “mesenchymal” and “epithelial” G-2 cell subpopulations, only the “epithelial” subpopulation formed lung metastases.

**Conclusions:**

We demonstrate the utility of our mouse model to analyze factors influencing tumor cell dissemination and metastasis. We suggest that the different metastatic capacity of G-2 and H8N8 cells is due to their different degrees of epithelial-mesenchymal plasticity (EMP), and thus the ability of the respective disseminated cells to revert from a “mesenchymal” to an “epithelial” differentiation state.

**Electronic supplementary material:**

The online version of this article (doi:10.1186/s12885-015-1165-5) contains supplementary material, which is available to authorized users.

## Background

Breast cancer is one of the most common cancers among women in developed countries, and about 16.7 percent of breast cancer patients die from the disease due to development of metastases [1]. Outgrowth of metastases may occur as late as 20 years after diagnosis and treatment, although several studies in mouse models suggest that cancer cell dissemination, the initial step of metastasis, can be a very early event in the disease [2-4]. In patients, the screening for and detection of circulating tumor cells (CTC) in blood samples and disseminated tumor cells (DTC) in bone marrow aspirates has become a valuable prognostic factor in patient care [5-9].

Understanding tumor cell dissemination in detail, and analyzing the fate of CTC and DTC up to the outgrowth of metastasis is an important task not only for further understanding subsequent steps of the metastatic cascade, but also for improving the diagnostic value of CTC and DTC for patients [10]. As experimental studies are very limited in humans, animal models are indispensable. So far, most studies are performed with xenograft models [11,12] which, however, face the problem that the influence of the immune system on various aspects of metastasis cannot be analyzed. Furthermore, and despite some similarities, the cellular environment of human and mouse cells may differ in important aspects. However, suitable immune competent mouse models to follow up metastasis formation from CTC and DTC are scarce.

In this study we analyzed tumor cell dissemination and metastasis in the WAP-T mouse model, a well characterized immune-competent mouse model for oncogene-induced mammary carcinogenesis. WAP-T mice [13,14] develop spontaneous mammary carcinomas upon induction via mating. Whey acidic protein (WAP) promoter dependent expression of SV40 T antigens leads to transformation of mammary epithelial cells and ultimately to tumor growth. Additional expression of mutant p53 in bi-transgenic WAP-T/WAP-mutp53 mice aggravates tumor progression, and enhances metastasis to the lungs [14]. The clinical relevance of the WAP-T mouse model is emphasized by comparison with human ductal carcinoma *in situ* [13,15] and molecular similarities between WAP-T and human triple-negative, basal-like and non-basal-like mammary carcinoma subtypes [16].

We succeeded in developing a WAP-T tumor cell line (G-2 cells), which reflects tumor cell heterogeneity and molecular characteristics of human breast carcinomas *in vitro* and *in vivo* after orthotopic transplantation into syngeneic WAP-T mice [17]. Due to an integrated, HA-tagged *mutp53* gene in G-2 cells, the transplantable WAP-T-G-2 tumor cell system allows analysis of tumor cell dissemination by a PCR assay [18]. As G-2 cell transplanted WAP-T mice so far failed to metastasize, we developed another WAP-T tumor cell line (H8N8 cells) with similar characteristics as G-2 cells, but with moderate metastatic capacity. We here describe the distribution and kinetics of tumor cell dissemination and of parameters influencing metastasis formation from DTC in WAP-T-NP8 mice transplanted with G-2 and H8N8 cells, respectively.

## Methods

### Animals

Mice were kept, bred, and handled under SPF conditions in the animal facility of the Heinrich-Pette-Institute as described previously [14,17] and approved by Hamburg’s Authority for Health (TVG 88/06, 34/08, 114/10, and 48/12). Orthotopic tumor cell transplantation was performed as described previously [17].

### Size of the animal cohorts used in this study

- *evaluation of metastasis rate in primary WAP-T tumors*: BALB/c: n = 39, T1: n = 86, NP8: n = 175; T1-H22: n = 28; NP8-H8: n = 40; NP8-W1: n = 32 and NP8-W10: n = 60.
- *tumor growth kinetics of transplanted G-2 and H8N8 cells*: NP8: n = 24.
- *detection of DTC/CTC in transplanted NP8 mice*: n = 23
- *detection of DTC/CTC in resected NP8 mice*: n = 37
- *immune system involvement for DTC/CTC frequency in transplanted mice*: NP8: n = 16, NSG: n = 27
- *experimental metastasis: serial dilution*: NP8: n = 48
- *experimental metastasis: time course*: NP8: n = 12

Except for the experiments involving endogenous tumor growth, all experiments were performed with at least two replicates.

### Cell culture

The WAP-T cell lines G-2 and H8N8 were cultured in DMEM medium (PAA) supplemented with 10% FCS (PAA) at 37°C and 5% CO_2_.

TGF-beta1 treatment: cells were treated 12 hours after seeding with 5 ng/ml TGF-beta1 (solubilized in 2 mg/ml BSA in PBS) purchased by R&D (#240-B-002/CF). Cells were harvested after 72 h incubation for further analysis.

### Histology

For histological analysis, lung specimen were processed as previously described [17]. Immunehistological stainings were performed with an home-made anti SV40 T-Ag rabbit polyclonal antibody (R15) [19] or a rabbit polyclonal anti HA-Tag (MBL-561).

### Immunofluorescence staining

Immunofluorescence staining was performed as described previously [17], see Additional file 1: Table S1. Secondary antibodies used for immunofluorescence staining were DyLight® or Alexa®Dye conjugates obtained from Invitrogen or Dianova.

### DNA extraction and PCR

DNA was extracted from blood and bone marrow after lysis of erythrocytes and from snap frozen tissues after homogenization with FastPrep by Phenol-Chloroform. For PCR analysis 200 ng of DNA was amplified with primers specific for the HA tag in the mutp53 expression cassette (forward GACCGCCGTACAGAAGAAGAA, reverse TCAGATCTTCAGGCGTAGTCG) using the 5′-Prime Taq-DNA polymerase kit. DNA extracted from cell lines or Balb/c mouse liver was used as controls. PCRs for the mouse *Notch4* gene were run in parallel (forward CTGCACCTAGCTGCCAGATTC and reverse CTGTCTGCTGGCCAATAGGAG).

### qPCR

RNA was purified using the Innuprep RNA-Extraction Kit (Analytik Jena) and reverse transcribed with the High Capacity RT kit (Applied Biosystems). PCR was performed using the Power SYBR Green PCR Mastermix (Applied Biosystems) in a standard program running in an ABI 7500 Fast thermal cycler (Applied Biosystems). PCR reactions for each sample were run in triplicate. See Additional file 1: Table S1 for the list of primers. *Hspa8* was used as housekeeping gene for sample normalization. Relative expression values for each gene were obtained through calculation of 2^–∆∆CT^ values, where ∆∆CT = delta delta CT values. Expression values of the mock samples were used as calibrator. Delta CT values were used for statistical analysis (Student’s *t*-test).

### Statistical analysis

All statistical analyzes were made with Graphpad Prism 5.0.

## Results

### The transplantable WAP-T mammary tumor model

*Mice, cell lines, and properties of transplanted tumors.* Mono-transgenic BALB/c WAP-T mice (lines WAP-T1, short T1; WAP-T-NP8, short NP8, [13]) and bi-transgenic Balb/c WAP-T x WAP-mutp53 mice (lines WAP-T1 x WAP-H22, short T1-H22; WAP-NP8 x WAP-W1, short NP8-W1; WAP-NP8 x WAP-W10, short NP8-W10 and WAP-NP8 x WAP-H8, short NP8-H8) develop invasive mammary carcinomas with roughly the same kinetics within 5–8 months, but differ significantly in their metastatic potential (Additional file 2: Figure S1A) [14,15]). To study metastatic processes in WAP-T tumors, we established clonal cell lines from a bi-transgenic T1-H22 tumor (G-2 cells and derivatives; [17]). G-2 cells, their clonal derivatives, and their properties in forming a self-reproducing mammary cancer cell system, have been described in detail [15,17]. Despite their origin from a bi-transgenic T1-H22 tumor, G-2 cells only weakly express mutp53 in cell culture as well as in transplanted tumors [15]. We so far did not observe metastasis when G-2 cells were orthotopically transplanted into WAP-T mice.

**Figure 1:**
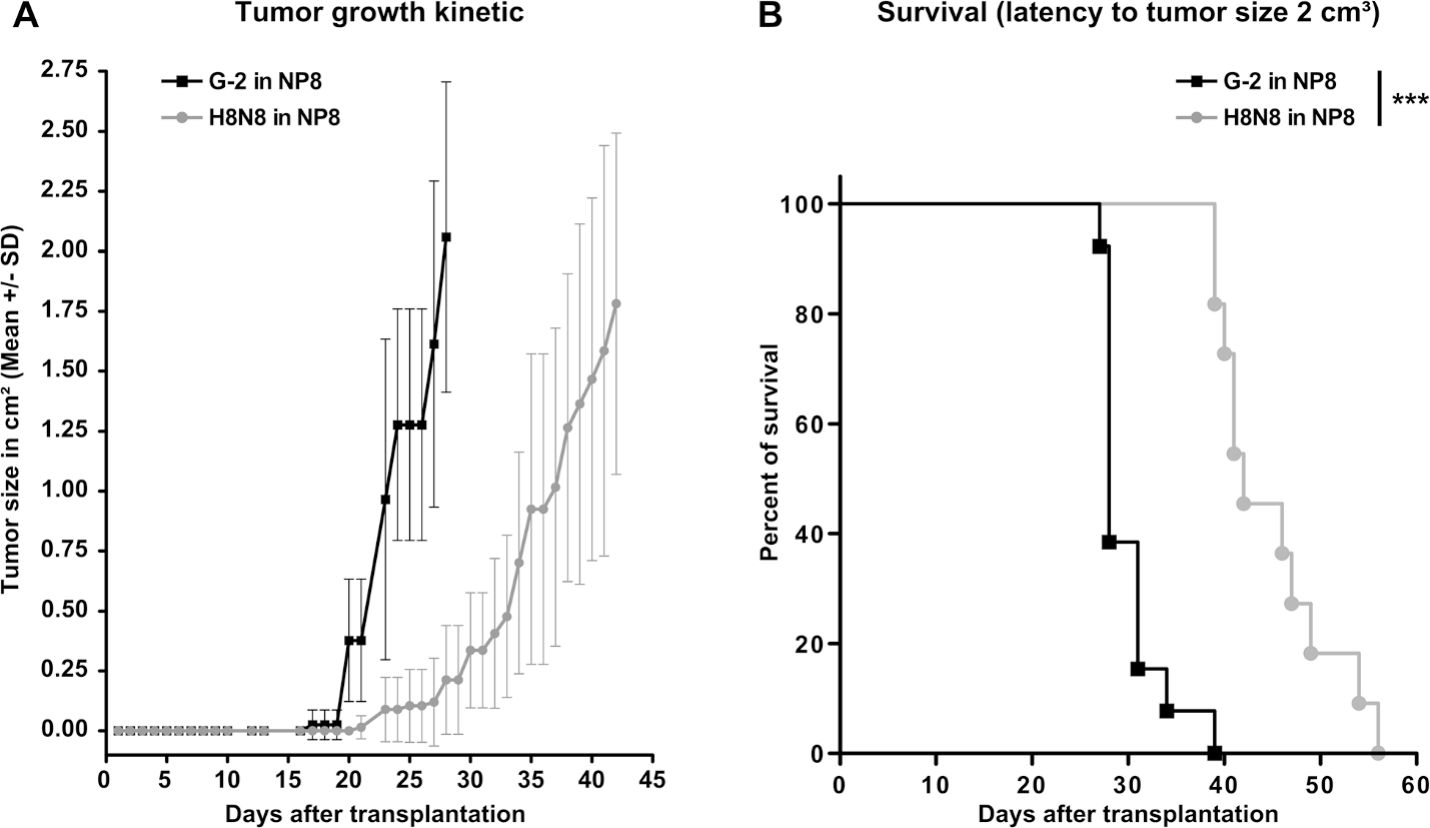
**Growth kinetics of WAP-T cell lines in NP8 recipient mice.** Tumor growth kinetics **(A)** and latency until sacrifice **(B)** in G-2 (n = 13) and H8N8 (n = 11) transplanted NP8 recipient mice. Female NP8 mice were orthotopically transplanted with 10^3^ G-2 or H8N8 cells into mammary gland #3 (abdominal left) and tumor growth was measured using a caliper twice per week. The median time for the growth of a 2 cm^3^ big tumor was 28 days and 42 days for G-2 and H8N8 cells, respectively (log-rank test p < 0.001).

We failed to establish similar cell lines from NP8-W1 and NP8-W10 mice. Similarly, it was not possible to establish such cell lines from 64 mono-transgenic T1 or NP8 tumors. For reasons unknown to us, it was only possible to develop G-2 like mammary carcinoma cell lines from bi-transgenic tumors containing the mutp53^R270H^ mutation (3 cell lines established out of 24 primary tumors), e.g. H8N8 cells established from a tumor of a bi-transgenic NP8-H8 mouse. H8N8 cells in culture show very similar properties as G-2 cells, but strongly express mutp53. Orthotopic transplantation of as few as 10 H8N8 cells also leads to mammary tumors of epithelial phenotype that show a much stronger and wider distribution of mutp53 expression than transplanted G-2 tumors (characterization of H8N8 *in vitro* as well as *in vivo* in supplemental data Additional file 3: Figure S2 and data not shown). G-2 cells transplanted NP8 mice showed an earlier onset of growth and a slightly faster tumor growth leading to a mean life time shortening of 14 days compared to mice transplanted with H8N8 cells (Figure 1). H8N8 tumors metastasized with a frequency of about 20% (Additional file 2: Figure S1B), while G-2 tumors failed to metastasize.

### DTC detection in transplanted NP8 mice

**Figure 2:**
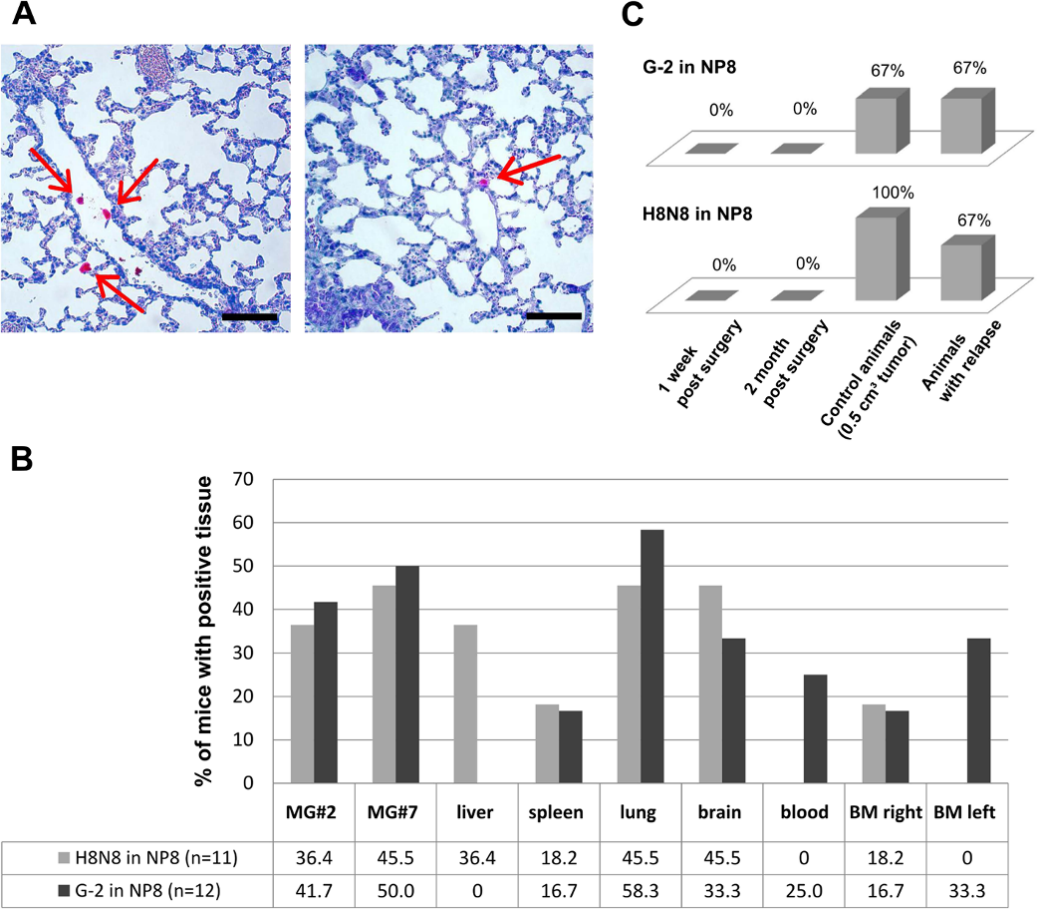
**Detection of DTCs in transplanted NP8 mice. (A)** Representative examples of serial lung tissue sections of mice carrying G-2 tumors at the time of sacrifice (tumor size 2 cm_3_), stained for T-Ag expression (red). Single positive cells (arrows) can be found in blood vessels and lung tissue. Scale bar = 200 μm. **(B)** Tumor cell dissemination in G-2 and H8N8 transplanted mice. NP8 mice were orthotopically transplanted with 10^3^ H8N8 cells (n = 11) or with G-2 cells (n = 12). Different mouse tissues, blood and bone marrow (BM) were analyzed by PCR for the occurrence of DTC (HA-signal) at the time of sacrifice (tumor size of 2 cm^3^). Plotted is the percentage of mice with positive signals in the respective tissue, blood or bone marrow. **(C)** Tumor cell dissemination in G-2 and H8N8 cell transplanted mice after tumor resection. NP8 mice were orthotopically transplanted with either 10^3^ H8N8 or G-2 cells. Tumor growth was monitored by caliper measuring. At 0.5 cm^3^ tumors were surgically removed and were sacrificed at 2 months (G-2: n = 5, H8N8: n = 5) and 1 week post surgery (G-2: n = 5, H8N8: n = 4). Animals with relapse (G-2: n = 6, H8N8: n = 3) and control mice (G-2: n = 3, H8N8: n = 6) were sacrificed at 0.5 cm^3^ tumor size. Different mouse tissues (mammary gland #7, liver, spleen, lung, brain), blood and bone marrow were analyzed by PCR for the occurrence of DTC (HA-signal). Plotted is the percentage of mice with positive signals in any of the analyzed tissues. Mice suffering a relapse of tumor growth are plotted separately.

Tumors and DTC of transplanted G-2 or H8N8 cells can be discriminated from non-tumor tissue of recipient NP8 mice by expression of SV40 T-Ag. Screening lungs of G-2 / H8N8 tumor bearing mice for the occurrence of metastases, occasional single T-Ag positive cells could be found (Figure 2A). For the analysis of tumor cell dissemination to different organs we established a genomic DNA based PCR which detects the specific HA-tag of the *mutp53* expression cassette in G-2 and H8N8 cell lines (for details see [15,18]). We determined the specificity of detection in BALB/c liver tissue to be in the range of 25 tumor cells in 1.000.000 tissue cells. To exclude the possibility that PCR detects free floating DNA we tested serum probes of several tumor-bearing animals for HA-DNA, and always obtained negative results (data not shown).

To estimate the distribution of DTC in various organs, we prepared genomic DNA from mammary gland #2 (MG#2), mammary gland #7 (MG#7), liver, spleen, lung, brain, blood and bone marrow (BM) of the right and the left femur of 11 NP8 mice transplanted with H8N8 cells and 12 NP8 mice transplanted with G-2 cells at the time of sacrifice with a tumor volume of approx. 2 cm^3^. We found DTC by PCR in every tissue with an average of 2–3 positive tissues per mouse. However, various tissues were not affected significantly different, as DTC were only slightly more often found in mammary glands, lungs and brain (Figure 2B). We did not detect HA-PCR signals in blood and left bone marrow of mice transplanted with H8N8 cells and no signals in liver of mice transplanted with G-2 cells. Altogether we conclude that neither G-2 nor H8N8 cells display a clear organ preference during dissemination. This was not necessarily to be expected as metastasis of primary WAP-T tumors in all our mouse lines is basically restricted to the lungs.

### Metastasis of disseminated tumor cells

Despite significant tumor cell dissemination into various organs from both, G-2 cell or H8N8 cell derived tumors, metastasis rates of the transplanted tumors were quite different for G-2 tumors (0%) compared to H8N8 tumors (~20%). We first asked whether this might reflect that G-2 cells are generally unable to colonize a target organ once they have entered the circulation, and performed experimental metastasis by intravenous (i.v.) injection of 10^5^ G-2 or H8N8 cells into the tail vein (TV) of NP8 mice. Tumor growth in the lungs occurred reproducibly for both cell lines. We lowered the numbers of TV injected cells down to 100, but did not find a significant difference between G-2 and H8N8 cells regarding the amount of cells needed in the circulation to initiate the development of lung metastases. It is estimated that a tumor of 1 cm^3^ sheds about 10^6^ tumor cells per day into the circulation [20]. Thus a 0.5 cm^3^ G-2 or H8N8 tumor would shed approximately 10^5^ cells per day. This should exclude that the quantity of tumor cells in the circulations limits metastasis of G-2 and H8N8 transplanted mice. As G-2 as well as H8N8 cells are able to colonize a target tissue with similar efficiency, and as DTC from their respective transplanted tumors are present in sufficient numbers, we assumed that the limited potential of DTC derived from G-2 tumors to form metastases has other reasons.

### Tumor cell dissemination and metastasis after tumor resection

Metastasis in breast cancer often is a rather late event in disease progression, occurring even 10 – 15 years after successful removal of the primary tumor. We, therefore reasoned that the lack of metastasis seen in G-2 transplanted mice, and the moderate metastasis rate observed in H8N8 transplanted mice might reflect the relatively short time of exposure to DTC. Mice after transplantation with G-2 cells only live approximately 28 days before they need to be sacrificed due to tumor burden. In contrast, mice transplanted with H8N8 cells display an extended life span of 42 days before tumors reach 2 cm^3^ (Figure 1A and B). In particular, endogenous primary tumors of WAP-T/WAP-mutp53 mice presumably have much more time for establishment and outgrowth of metastases (about 200 days after trangene induction for NP8, NP8-W1 and NP8-W10 mice), as early tumor cell dissemination is a well known phenomenon [2,3].

Mimicking the clinical situation we resected transplanted G-2 and H8N8 tumors when they reached a palpable size (0.5 cm^3^, at approx. 20 days for G-2 transplanted and approx. 30 days for H8N8 transplanted tumors) and analyzed dissemination and metastasis at different time points thereafter (1 week, 2 months). Control animals were sacrificed at a tumor size of 0.5 cm^3^. At this time point, on average 70% of G-2 cell and 100% of H8N8 cell transplanted mice presented with HA-tag-positive tissues (Figure 2C). Tumor resection in our experimental system led to a drop in DTC frequency below detection limit already one week post-surgery (the first time point analyzed) and from then on. DTC frequency went back to pre-surgery levels in animals that suffered a relapse. We did not observe metastases in G-2 and H8N8 transplanted mice where tumors were successfully resected. Single transplanted mice were left alive up to 8 months post-surgery without development of metastases or relapse. We conclude that levels of disseminated G-2 and H8N8 cells are maintained by continuous cell shedding from the tumor. However, the vast majority of disseminated G-2 and H8N8 cells cannot survive and proliferate in their target tissues. Mice thus are exposed to detectable levels of DTC only during tumor growth. The short half-life of DTC in our system explains, why no metastases were found in H8N8 transplanted mice, when the tumors were resected at a tumor size of 0.5 cm^3^, whereas about 20% of H8N8 cell transplanted tumors metastasized when the tumors were allowed to grow up to a volume of 2 cm^3^. We conclude that metastatic outgrowth of H8N8 DTC is a rare stochastic event, whose probability is enhanced the longer the animals are exposed to DTC. It was not possible to test, whether longer exposure of G-2 cell transplanted NP8 mice to DTC would lead to metastasis, as mice have to be sacrificed at a maximal tumor volume of 2 cm^3^ due to ethical reasons. Furthermore, alternate parameters that limit metastasis in G-2 transplanted mice should be considered, like immunological elimination or apoptosis of circulating cells before they reach the target organ, or a poor ability to colonize the respective target organ.

### Fate of G-2 cells in experimental metastasis

**Figure 3:**
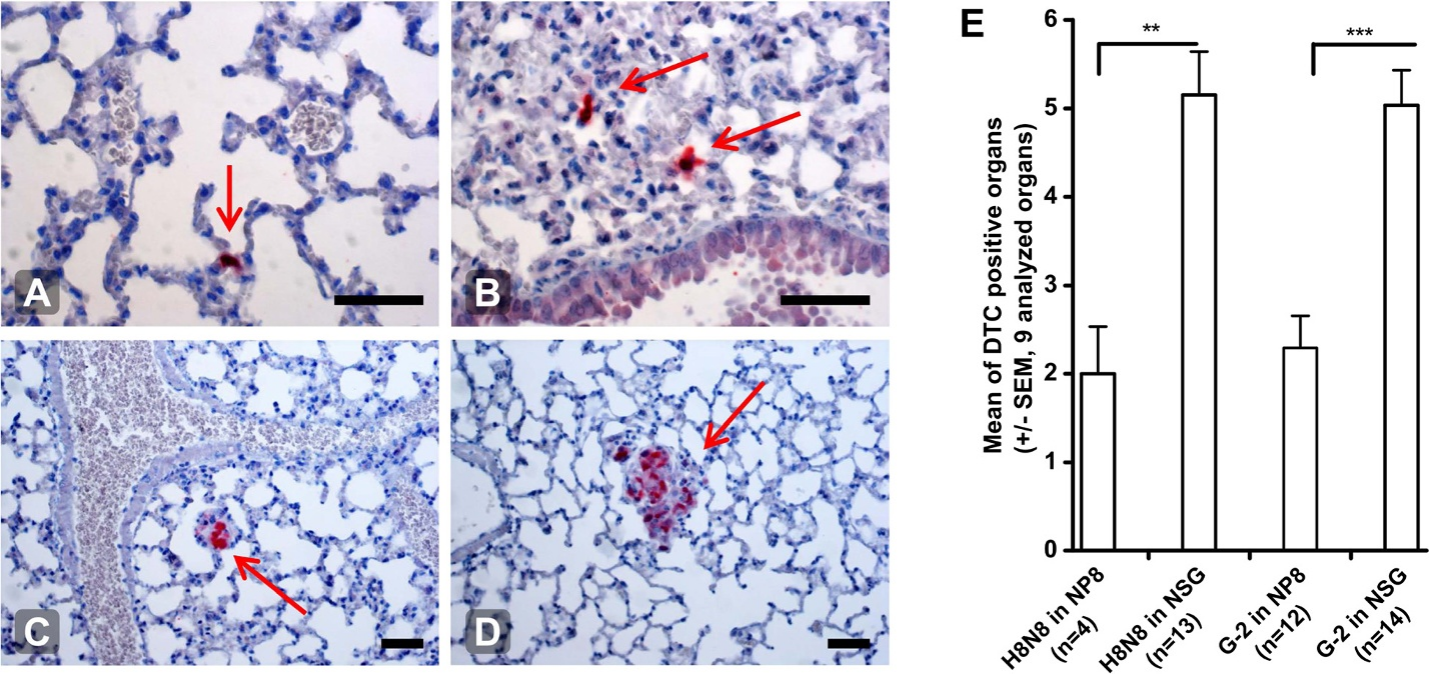
**Experimental metastasis and influence of the immune system.** Fate of TV injected tumor cells. Lung sections of mice, TV injected with 10^5^ G-2 cells and sacrificed at 1 h, 1 d, 1 week and 2 weeks post injection, stained for T-Ag expression (red; arrows). **(A)** and **(B)** 1 h post injection: cells have left the circulation and entered lung tissue, 20× magnification; **(C)** micrometastasis 1 week post injection; **(D)** metastasis 2 weeks post injection, 10× magnification. Scale bars = 100 μm **(E)** Tumor cell dissemination in immune deficient mice. NP8 and NSG mice were orthotopically transplanted with either 10^3^ H8N8 or G-2 cells (H8N8 in NP8 n = 4, H8N8 in NSG n = 13, G-2 in NP8 n = 12 and G-2 in NSG n = 14). Mice were sacrificed at a tumor volume of 2 cm^3^ and different mouse organs were analyzed by PCR for the occurrence of DTC (HA-tag signal): mammary gland #2, #7, liver, spleen, lung, brain, blood, bone marrow left and right. In NP8 mice on average 2 out of 9 tissues were positive for DTC and in NSG mice 5 out of 9. Statistical analysis: unpaired *t* test. ** p = 0.0019, *** p < 0.0001.

**Figure 4:**
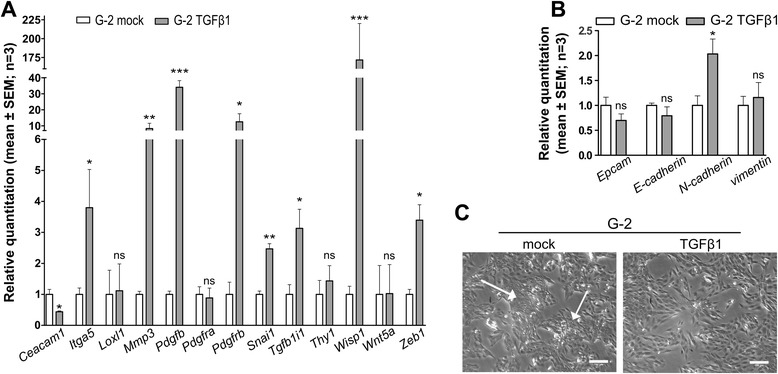
**TGFß1 induced epithelial-mesenchymal plasticity (EMP) in G-2 cells.** G-2 cells were treated with TGFβ1 (7.5 ng/ml) for 72 h. Relative quantitation of **(A)** EMT signature gene expression and **(B)** epithelial and mesenchymal markers expression was performed via RT-qPCR in mock- and TGFβ1-treated cells (n = 3 replicates). *Hspa8* was used as a housekeeping gene for sample normalization. Relative expression values for each gene were obtained through calculation of 2^–∆∆CT^ values, where ∆∆CT = delta delta CT values. Expression values of the mock samples were used as calibrator. Delta CT values were used for statistical analysis (Student’s *t*-test). **(C)** Phase contrast images of either mock- or TGFβ1-treated G-2 cells. The white arrows show dense colonies of epithelial cells in untreated G-2 cell cultures; scale bar: 150 μm.

We next used TV injected G-2 cells as a model for disseminated G-2 cells to have a closer look at their fate after inoculation into the circulation. Mouse lungs were prepared 1 h, 1 day, 1 week and 2 weeks after initiation of experimental metastasis with 10^5^ G-2 cells. Lungs were paraffin-embedded and serial sections stained for SV40 T-Ag by immunohistochemistry (Figure 3A-3D). 1 h after TV injection between 2 to 12 single G-2 cells were visible in lung tissue on each analyzed section. In parallel, we performed HA-tag-specific PCRs on different tissues of the same mice (mammary glands 2, 3, 7, liver, spleen, lung and blood) (Additional file 2: Figure S1C). 1 h post injection the bulk of the signal was found in the lungs only. One mouse showed a weak signal in the spleen and another a very weak signal in mammary gland #2. Assuming an equal distribution of tumor cells within the lung, we calculated that 1 h after injection about a quarter of the TV injected G-2 cells could be detected by immunohistochemistry in the lungs. Besides intact tumor cells, we already at this time point found tumor cell debris in the lung. Remarkably, no tumor cells or tumor cell debris were seen in blood vessels (Figure 4A). On day 1 after TV injection we found a major drop in SV40 T-Ag positive cells in lung tissue. Most sections did not contain any tumor cells anymore, but each mouse harbored one or two sections out of 6 analyzed with a single tumor cell. These cells were still detectable by HA-specific PCR, though the signals were weaker. Already 1 week post TV injection 2 out of 3 mice harbored several micro-metastases (<10 cells in diameter) or metastases. But single T-Ag positive cells were no longer visible. 2 weeks post TV injection, 1 out of 3 mice showed micro- and metastasis in every lung section. Thus half the mice injected with 10^5^ G-2 cells developed metastases within 1–2 weeks.

We conclude that circulating G-2 cells leave the blood circulation within the first hour after injection to invade adjacent tissue. Thereafter, the majority of cells fails to proliferate and cells are eliminated. Injected with the same amount of cells, some mice develop several lung tumors, whereas other mice obviously are able to clear G-2 cells. Only rarely a few dormant cells survive for longer periods of time. Such rare dormant cells were also occasionally observed in G-2 and H8N8 transplanted mice that did not develop tumors up to 6 months post transplantation (data not shown).

### Influence of the immune system on tumor cell dissemination and metastasis

In order to find out if an immune reaction might impair tumor cell dissemination and metastasis, we transplanted G-2 and H8N8 cells into immune-competent NP8, and into immune-deficient NOD scid gamma (NSG) mice. Primary tumor growth did not significantly differ between NP8 and NSG mice. However, tumor cell dissemination was significantly stronger in NSG mice for both, H8N8 and G-2 cell transplanted mice, with an average of 5 PCR-positive tissues out of 9 tissues analyzed (Figure 3E) compared to NP8 mice (approx. 2 of 9 tissues analyzed). Interestingly, the rate of metastasis of H8N8 cells in NSG mice increased to 40% (5 out of 13 mice, Additional file 2: Figure S1B), while no metastasis could be found in NSG mice transplanted with G-2 cells. We conclude that in immune-competent mice primary tumor growth is not affected by the immune system, whereas a so far undefined immune reaction corroborates the extent of tumor cell dissemination. In the case of H8N8 tumors, the absence of a functional immune system led to enhanced metastasis, possibly corresponding to enhanced tumor cell dissemination. In contrast, no influence of the immune system and of enhanced tumor cell dissemination could be observed on metastasis of G-2 cell transplanted mice. We conclude that disseminated G-2 cells must lack an intrinsic property necessary to allow colonization of a respective target organ.

### Epithelial-mesenchymal plasticity (EMP) is possibly the decisive feature for metastatic outgrowth of disseminated WAP-T tumor cells

#### The EMP phenotype is independent from the morphological tumor cell phenotype

The ability of tumor cells to reversibly undergo epithelial to mesenchymal transition (EMT) and the reverse differentiation process MET (mesenchymal-epithelial transition) has been termed epithelial-mesenchymal plasticity (EMP) [21,22], and is an important feature of metastatic tumor cells. We recently compared the gene expression profiles of WAP-T/WAP-mutp53 bi-transgenic tumors and of NP8 tumors, and identified a mutp53-induced ‘EMT gene signature’ [15]. Despite an enhanced expression of genes associated with the oncogenic EMT gene network in bi-transgenic tumors, mono- and bi-transgenic tumors showed an indistinguishable histology, indicating phenotypic plasticity, i.e. an EMP-phenotype of WAP-T tumors cells.

To get further experimental support for this assumption, we analyzed the phenotypic conversion of G-2 cells in culture after application of the well-known EMT-inducer TGFß1 [23]. TGFß1 also is a major factor of the tumor microenvironment in WAP-T tumors [15]. As expected, a 3 day TGFß1 treatment induced a change in cell morphology (Figure 4C), as G-2 cells almost completely lost their epithelial cell compartment, which is typically organized in dense colonies (white arrows, Figure 4C). Instead, treated G-2 cells now all displayed a more homogenous, elongated, spindle-like morphology which is characteristic for cells that have undergone EMT. Furthermore, we tested the cells for the expression of EMT signature genes and for the expression of phenotypic epithelial and mesenchymal markers. 9 out of the 14 genes of the ‘EMT gene signature’ were significantly regulated (Figure 4A). However, concerning the expression of phenotypic epithelial and mesenchymal markers, we observed only regulation of *N-cadherin*, while expression levels of *EpCAM*, *E-cadherin* and *vimentin* did not change significantly (Figure 4B). Thus treatment of G-2 cells with the potent EMT-inducer TGFβ1 induced an enhanced plasticity of the tumor cells rather than a complete EMT, as evidenced by the absence of changes in the levels of phenotypic markers.

This led us to propose that the ability of DTC to colonize a target organ most likely is more dependent on their EMP properties than on their morphological phenotype. EMP properties are required to quickly reverse from a ‘quasi-mesenchymal’ to a quasi-epithelial’ phenotype once DTC enter a target organ.

#### Experimental metastasis of EpCAM-sorted G-2 cells

G-2 cells, like H8N8 cells were able to efficiently colonize the lungs in experimental metastasis after TV injection. In these experiments cells derived from cell culture were injected. In culture, these cells comprise a homeostatic mixture of ‘quasi-mesenchymal’ and ‘quasi-epithelial’ cells, i.e. of cells in states differing in their degree of EMP [17]. We thus considered the possibility that these two cell compartments might differ in their metastatic capacity, and performed experimental metastasis with FACS pre-sorted G-2 cells. 5 × 10^4^ EpCAM^high^, 5 × 10^4^ EpCAM^low^ and 5 × 10^4^ EpCAM^high/low^ mixed cells were TV injected into NP8 mice and metastasis of the lungs analyzed after 6 weeks. 2 out of 9 mice injected with G-2-EpCAM^high^ cells, and 2 out of 9 mice injected with G-2-EpCAM^high/low^ mixed cells, but none of the 8 mice injected with G-2-EpCAM^low^ cells developed metastasis. Thus G-2 cells expressing the epithelial differentiation marker EpCAM were more successful in establishing metastasis than cells of a more mesenchymal differentiation state. A possible explanation of this result could be that only G-2 cells in the EpCAM^high^ population are in an EMP-state that allows colonization of the lungs.

## Discussion

In this study we used two tumor cell lines, G-2 and H8N8, to study tumor cell dissemination and metastasis from tumors arising in immune-competent syngeneic NP8 mice. G-2 and H8N8 cells exhibit very similar properties in cell culture and form tumors with high histological and molecular similarity to endogenous undifferentiated tumors [17,24]. As these tumors could be cross-species validated with corresponding triple-negative human tumors [16,24], the transplantable WAP-T tumor model constitutes a valuable tool for analyzing various aspects of tumor metastasis. Both cell lines were developed from bi-transgenic WAP-T/WAP-mutp53 tumors carrying a *mutp53* minigene with the R270H mutation (corresponding to the human (R273H) mutation. Why in the WAP-T system only mutp53^R270H^ acted as survival factor for *in vitro* culture of tumor cells is a not understood, but interesting phenomenon. However, a pro-survival function *in vitro* by inhibiting apoptosis has been described for several mutp53 proteins, including mutp53^R273H^ [25,26]. Such a pro-survival function of mutp53^R270H^ might confer a growth advantage to primary tumor cells in culture, thereby facilitating their establishment as a cell line.

While we so far failed to observe metastasis from G-2 transplanted NP8 mice, H8N8 mice metastasize with a moderate frequency of about 20%. It is interesting that expression of the transgenic mutp53^R270H^ in G-2 cells is rather weak and confined to single cells, while expression of mutp53^R270H^ in H8N8 cells is strong, both *in vitro* and in tumors. Whether this is only a corollary, or is causative, remains to be investigated.

Despite the difference in metastatic capacity, tumor cell dissemination was rather similar from tumors arising from both cell lines, and affected a variety of organs. This was somewhat unexpected, as the vast majority of metastases observed from endogenous tumors are found in the lung. This implies, that organ tropism of metastasis, at least in the WAP-T tumor system, is not decided at the level of tumor cell dissemination, but rather at the level of organ colonization.

To get clues for the different metastatic capacity of disseminated H8N8 and G-2 cells, respectively, we first excluded that G-2 cells are generally unable to colonize a target organ and performed experimental metastasis by TV injection of G-2 and H8N8 cells into NP8 mice. Surprisingly, even very low numbers of TV injected G-2 as well as of H8N8 cells were able to form tumors in the lungs, indicating that under this experimental setting cells from both lines are able to leave the circulation and build up metastases in a target organ with similar efficiency.

In analogy to the human situation, where metastasis is a rather late event in disease progression, we resected the transplanted tumors at a rather small tumor volume to provide a longer time of exposure to DTC for the development of metastases. Neither H8N8, nor G-2 cell transplanted mice developed metastases, and mice, from which tumors had been successfully removed, were cured. This finding might be important for assigning the metastatic capacity of tumors in tumor models relating to the human situation [27]. Parallel analysis for the presence of DTC in tumor resected mice revealed that DTC no longer could be detected already one week after tumor resection (the earliest time point analyzed). The lack of metastases in H8N8 cell tumor resected mice indicates that metastatic outgrowth of a disseminated H8N8 cell is a rather rare event, which requires the continuous presence of the short-lived DTC over a longer period of time. Experimental metastasis allowed analysis of the fate of G-2 cells once they reach the blood circulation. Interestingly, inoculated G-2 cells left the circulation within the first hour, and about a quarter of the cells reached the lungs as target organ. In accordance with our tumor resection data, most of the cells were eliminated rather fast, and only few cells survived and were able to build up a metastatic lesion.

We also compared tumor cell dissemination and metastasis from G-2 and H8N8 transplanted tumors in NP8 and in NSG mice. Transplanted NSG mice showed a significantly higher rate of tumor cell dissemination. In the case of H8N8 cells this led to a higher rate of metastasis, in accordance with our interpretation that enhanced or prolonged exposure of mice to disseminated H8N8 cells enhanced the probability for metastatic outgrowth. The reason for the enhanced dissemination in NSG mice is not known to us and its elucidation would require more detailed analyses.

To resolve the apparent discrepancy between metastatic efficiency of G-2 cells in experimental and in’real’ metastasis, we followed up on our recent data showing that EMP is a decisive factor for metastasis of WAP-T tumor cells [15]. Although G-2 cells do not lack EMP properties, as shown by treatment of G-2 cells in culture with TGFß1, only the EpCAM^high^ and the mixed population were able to form metastases after TV injection, when cultured G-2 cells were separated into an EpCAM^high^ and an EpCAM^low^ population. There is evidence that efficient metastasis requires re-differentiation (MET) [28,29]. While cells featuring EMT characteristics are by far more prone to disseminate from the primary tumor [30,31], epithelial cell characteristics are associated with a dramatic increase in colonization of the secondary site [28]. We previously showed that *in vitro* cells of the EpCAM^low^ G-2 population are in a mesenchymal differentiation state which does not allow a rapid conversion to the epithelial phenotype. [17]. Such conversion, however, is required for successfully building up a viable cancer cell system in the target organ. In contrast, the differentiation state of EpCAM^high^ cells seems to facilitate such conversion. With regard to the inability of disseminated G-2 cells to metastasize, this would imply that they are in a differentiation state which resembles that of the EpCAM^low^ G-2 population in culture.

## Conclusions

The present results have potential clinical implications. Large-scale meta-analyses have shown that the presence of DTCs is associated with an increased risk of relapse and shorter survival [32]. However, many patients with DTCs do not experience relapse within 10 years after, indicating that only a subset of DTC may have the ability to outgrow into an overt metastasis [32]. To further understand which DTC subset is metastatic, it will be necessary to identify the factors contributing to metastatic outgrowth. The transplantable G-2/H8N8 WAP-T tumor cell system described here might help to elucidate some of the requirements necessary for a DTC to successfully undergo the last steps in metastasis – the survival and proliferation in the target organ for metastasis.

## Additional files

Additional file 1: Table S1. Material list.
Additional file 2: Figure S1. Metastasis of primary WAP-T tumors and transplanted tumors of G-2 and H8N8 cells.
Additional file 3: Figure S2. Immunofluorescence characterization of H8N8 cells.

## Abbreviations

BM: Bone marrow
BSA: Bovine serum albumin
cm^3^: Cubic centimeter
CTC: Circulating tumor cells
d: Day/s
DMEM: Dulbecco’s modefied Eagle medium
DTC: Disseminated tumor cells
e.g.: For example
EMP: Epithelial-mesenchymal plasticity
EMT: Epithelial-mesenchymal transition
EpCAM: Epithelial cell adhesion molecule
FCS: Fetal calf serum
h: Hour
HA: Human influenza hemagglutinin
i.e.: That is
i.v.: Intravenous
MET: Mesenchymal-epithelial transition
MG: Mammary gland
mutp53: Mutant protein 53
NSG: NOD/scid gamma
PBS: Phosphate-buffered solution
SV40: Simian virus 40
T-Ag: T-antigen
TGFß1: Transforming growth factor beta 1
TV: Tail vain
WAP: Whey acidic promoter
