# Supplementary material for: Epithelial-mesenchymal plasticity is a decisive feature for the metastatic outgrowth of disseminated WAP-T mouse mammary carcinoma cells
Source: BMC Cancer. 2015 Mar 26;15:178. doi: 10.1186/s12885-015-1165-5 (PMC4381675; doi:10.1186/s12885-015-1165-5)
Supplement: Additional file 1: Table S1. — Material list. [file 12885_2015_1165_MOESM1_ESM.pdf]

Table S1: material list

Primers for quantitative PCR

Sequence	Name	Orientation	Gene
CGGCTGCGAGAGAAATTGC	Vim-Q1	sense	vimentin
CCACTTTCCGTTCAAGGTCAAG	Vim-Q2	antisense	
GAGTCCGAAGAACCGACAAGG	Epcam-Q1	sense	epithelial cell adhesion molecule
CTGATGGTCGTAGGGGCTTTC	Epcam-Q2	antisense	
GAATCCAGTCAGCGTCAGGAG	mCC1-Q1a	sense	Carcinoembryonic antigen-related cell adhesion molecule 1
CCGCCAGACTTCCTGGAATAG	mCC1-Q2a	antisense	
CCACCATGTCTACGAGCTCATC	Itga5-_Q1	sense	Integrin alpha-5
GGTGTAGTTGGAGGTGCAGTTG	Itga5-_Q2	antisense	
GACTGCCAGTGGATCGACATA	Loxl1-Q1	sense	Lysyl oxidase-like 1
CCACGTTGTTGGTGAAGTCAG	Loxl1-Q2	antisense	
TGTCCCGTTTCCATCTCTCTC	Mmp3-Q1	sense	matrix metalloproteinase-3
TGGTGATGTCTCAGGTTCCAG	Mmp3-Q2	antisense	
AAGCCTGACGTTGCTGATGA	Pdgfrb-Q1	sense	Beta-type platelet-derived growth factor receptor
TCTGCTTGCTGTGGCTCTTC	Pdgfrb-Q2	antisense	
CGCTGACAGTGGCTACATCAT	Pdgfra-Q1	sense	Alpha-type platelet-derived growth factor receptor
AACCTGTCTCGATGGCACTCT	Pdgfra-Q2	antisense	
GCTGCAATAACCGCAATGTG	Pdgfb-Q1	sense	Platelet-derived growth factor subunit B
CACTGTGGCCTTCTTGAAGATG	Pdgfb-Q2	antisense	
CTGGTGAGAAGCCATTCTCCT	Snai1-Q1	sense	Zinc finger protein SNAI1
CCTGGCACTGGTATCTCTTCA	Snai1-Q2	antisense	
CCTCTGTGGCTCCTGCAATA	Tgfb1i1-Q1	sense	Transforming growth factor beta-1-induced transcript 1
AGCGCTCAAAGTAGCACTCG	Tgfb1i1-Q2	antisense	
GAACTCTTGGCACCATGAACC	Thy1-Qa1	sense	Thymocyte antigen 1
GTTATTCTCATGGCGGCAGTC	Thy1-Qa2	antisense	
ACAGAGGCTGCCATCTGTGAC	Wisp1-Q1	sense	WNT1-inducible-signaling pathway protein 1
CTCGCCATTGGTGTAGCGTA	Wisp1-Q2	antisense	
GGAACGAATCCACGCTAAGG	Wnt5a-Qa1	sense	Wnt5a
GGAGCCAGACACTCCATGAC	Wnt5a-Qa2	antisense	
CACCAGAAGCCAGCAGTCAT	Zeb1-for	sense	Zinc finger E-box binding homeobox 1
CGTTCTTCTCATGGCGGTACT	Zeb1-rev	antisense	
GCGCAGTCTTACCGAAGGATG	Cdh2-Qa1	sense	N-cadherin
ATACACCGTGCCGTCCTCGT	Cdh2-Qa2	antisense	
AGCCATTGCCAAGTACATCCTC	E-Cdh-Q1	sense	E-cadherin
GGCCTGTTGTCATTCTGATCTG	E-Cdh-Q2	antisense	
CCGATGAAGCTGTTGCCTAT	qRT-Hspa8-3	sense	Heat shock 70 kDa protein 8
GTGACATCCAAGAGCAGCAA	qRT-Hspa8-4	antisense	

List of Antibodies

Antibody	Spezies	Method	Dilution	Company (Cat. No.)
HA (3F10)	rat	IF	1:500	Roche (11867423001)
Epcam (G8.8)	rat	IF	1:100	BioLegend (118201)
Vimentin (C20)	goat	IF	1:100	Santa Cruz (sc-7557)
HA-tag	rabbit	IHC	1:300	MBL (MBL-561)
SV40-LT	rabbit	IF, IHC	1:500	home made
K8/18	guinea pig	IF	1:200	Acris (BP5007)
K14	rabbit	IF	1:500	Covance (PRB-155P)

Melting curves of the respective primer paires

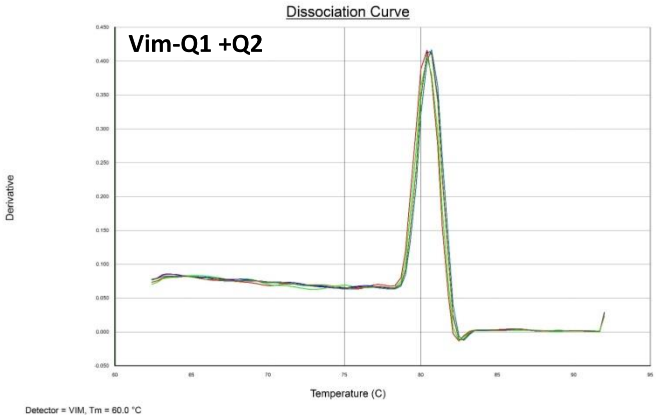

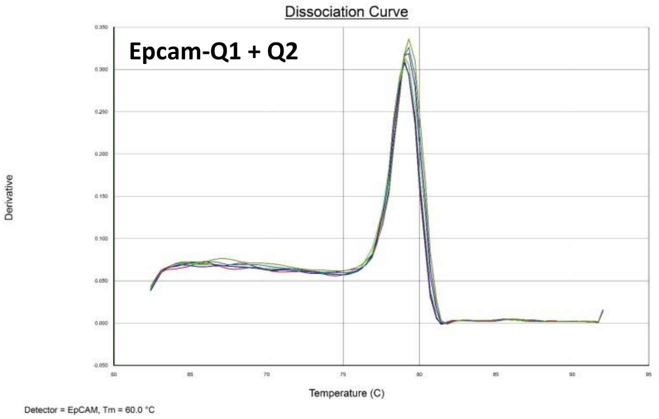

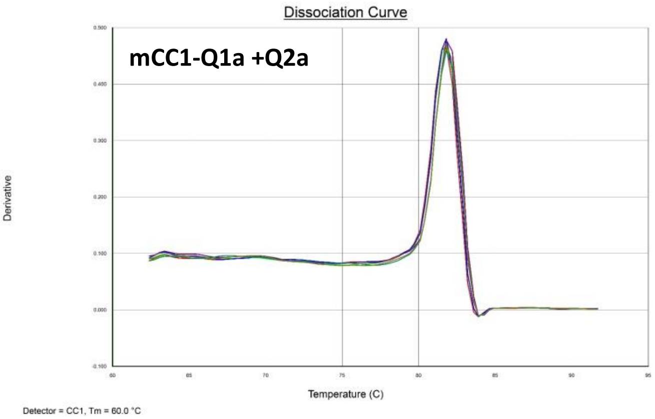

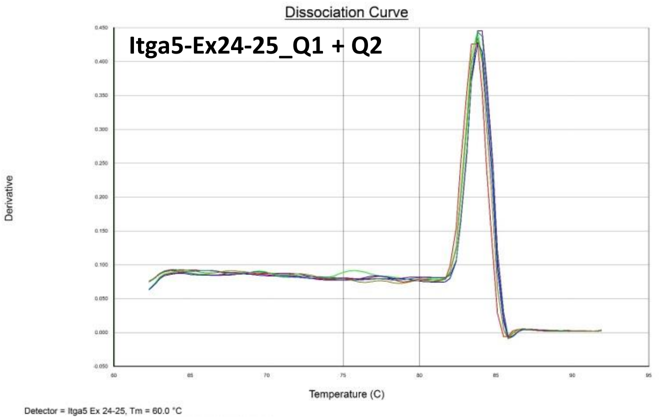

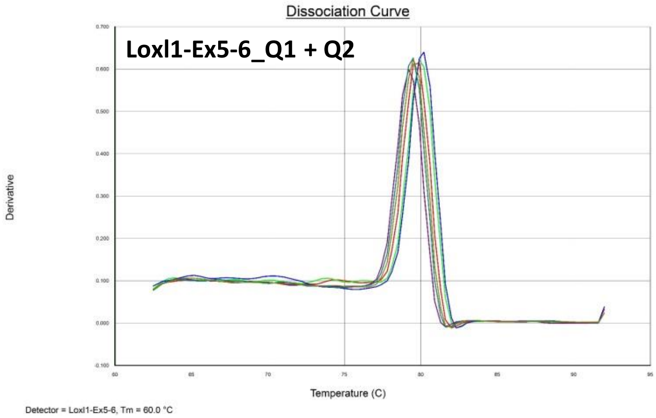
Dissociation Curve
Loxl1-Ex5-6_Q1 + Q2
Derivative
Temperature (C)
Detector = Loxl1-Ex5-6, Tm = 60.0 °C

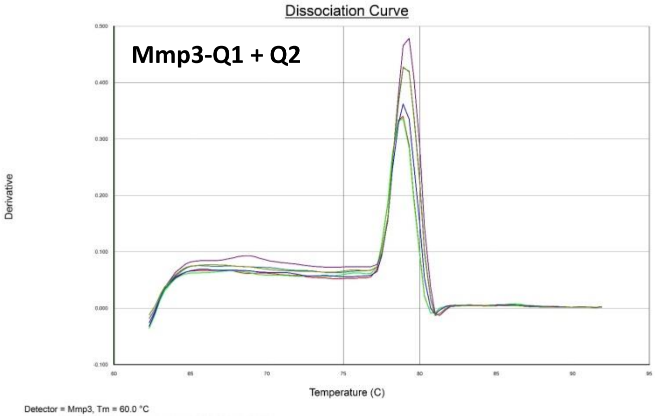
Dissociation Curve
Mmp3-Q1 + Q2
Derivative
Temperature (C)
Detector = Mmp3, Tm = 60.0 °C

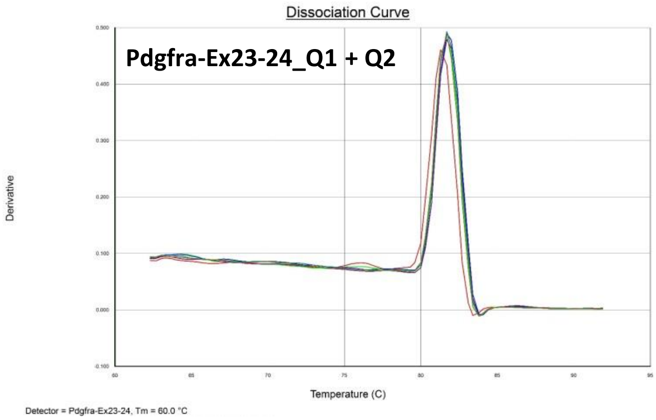
Dissociation Curve
Pdgfra-Ex23-24_Q1 + Q2
Derivative
Temperature (C)
Detector = Pdgfra-Ex23-24, Tm = 60.0 °C

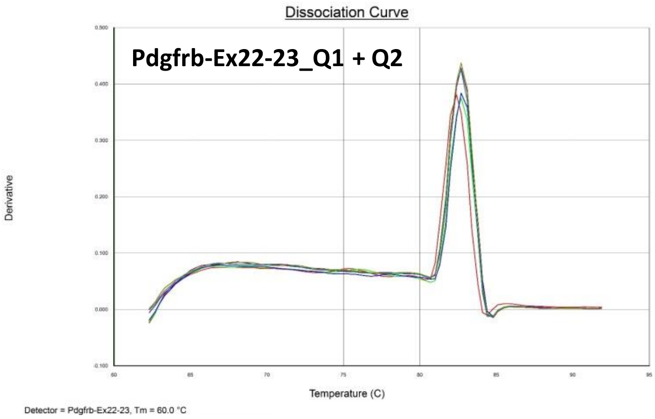
Dissociation Curve
Pdgfrb-Ex22-23_Q1 + Q2
Derivative
Temperature (C)
Detector = Pdgfrb-Ex22-23, Tm = 60.0 °C

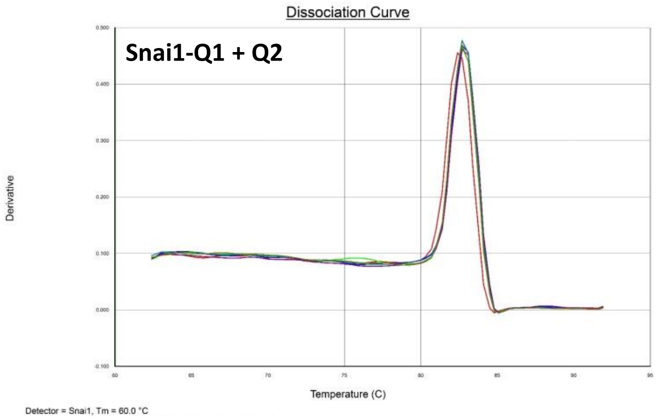
Dissociation Curve
Snai1-Q1 + Q2
Derivative
Temperature (C)
Detector = Snai1, Tm = 60.0 °C

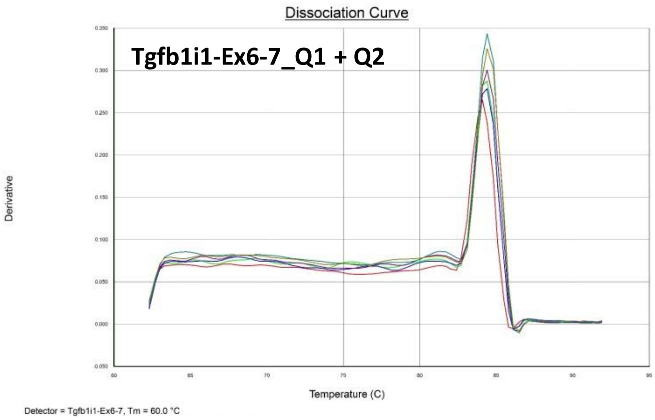
Dissociation Curve
Tgfb1i1-Ex6-7_Q1 + Q2
Derivative
Temperature (C)
Detector = Tgfb1i1-Ex6-7, Tm = 60.0 °C

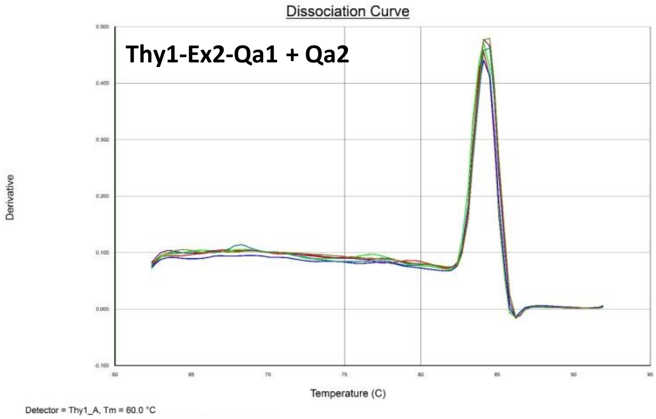
Dissociation Curve
Thy1-Ex2-Qa1 + Qa2
Derivative
Temperature (C)
Detector = Thy1_A, Tm = 60.0 °C

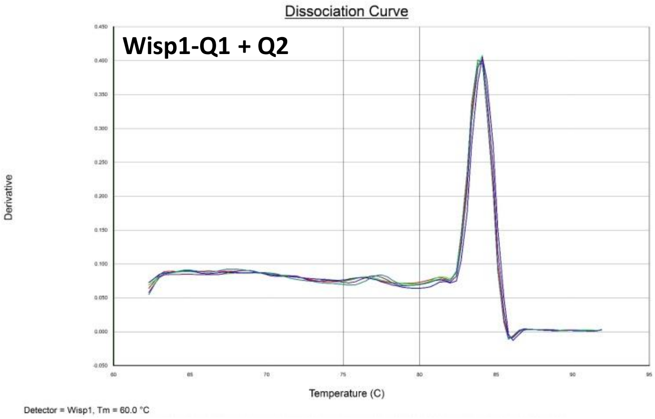
Dissociation Curve
Wisp1-Q1 + Q2
Derivative
Temperature (C)
Detector = Wisp1, Tm = 60.0 °C

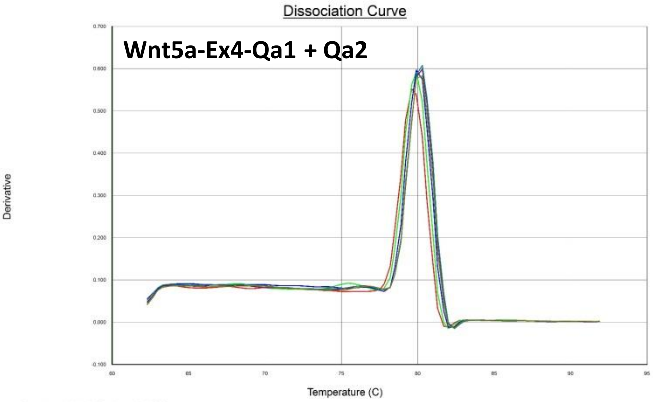

Detector = Wnt5a-Qa, Tm = 60.0 °C

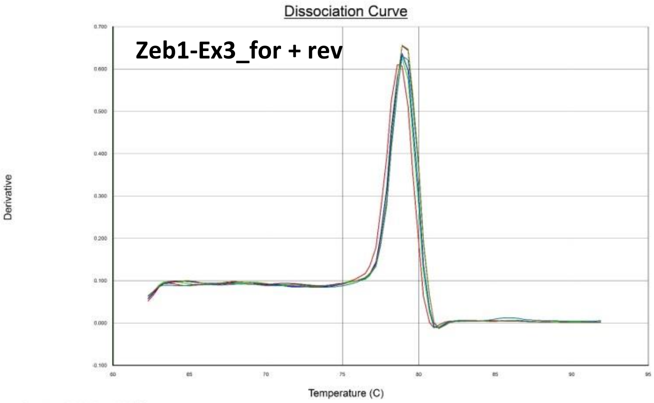

Detector = Zeb1, Tm = 60.0 °C

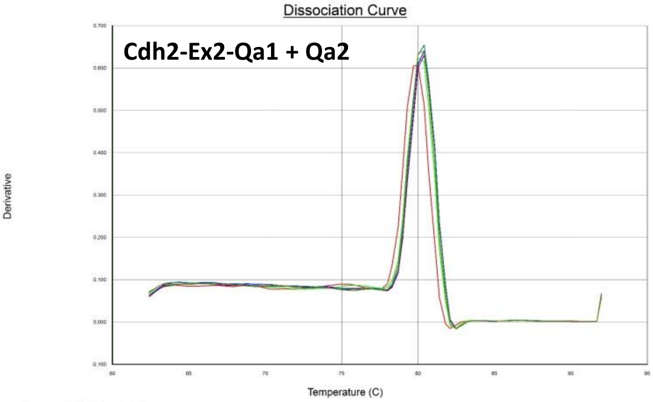

Detector = N-Cdh, Tm = 60.0 °C

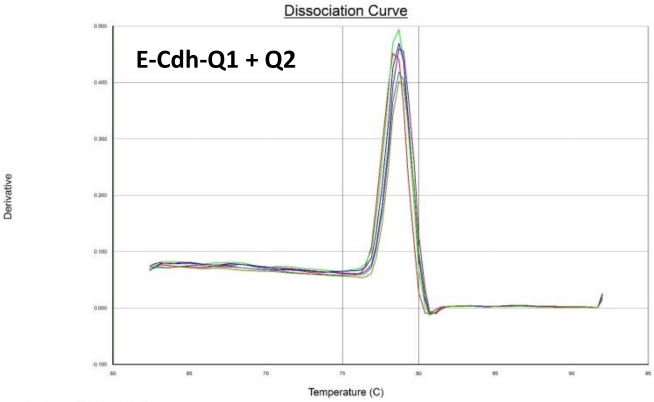

Detector = E-Cdh, Tm = 60.0 °C

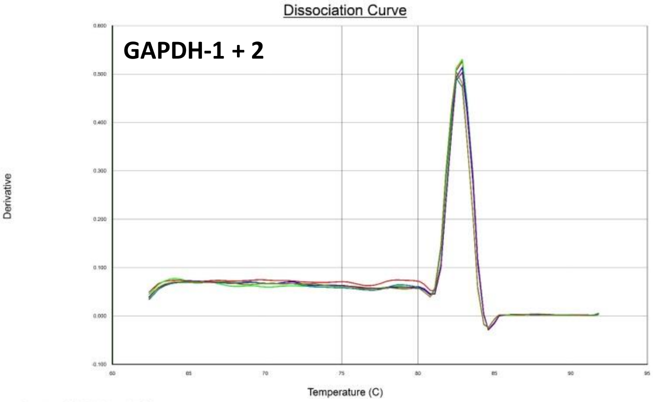

Detector = GAPDH, Tm = 60.0 °C
