# Supplementary material for: Epithelial-mesenchymal plasticity is a decisive feature for the metastatic outgrowth of disseminated WAP-T mouse mammary carcinoma cells
Source: BMC Cancer. 2015 Mar 26;15:178. doi: 10.1186/s12885-015-1165-5 (PMC4381675; doi:10.1186/s12885-015-1165-5)
Supplement: Additional file 2: Figure S1. — Metastasis of primary WAP-T tumors and transplanted tumors of G-2 and H8N8 cells. [file 12885_2015_1165_MOESM2_ESM.pdf]

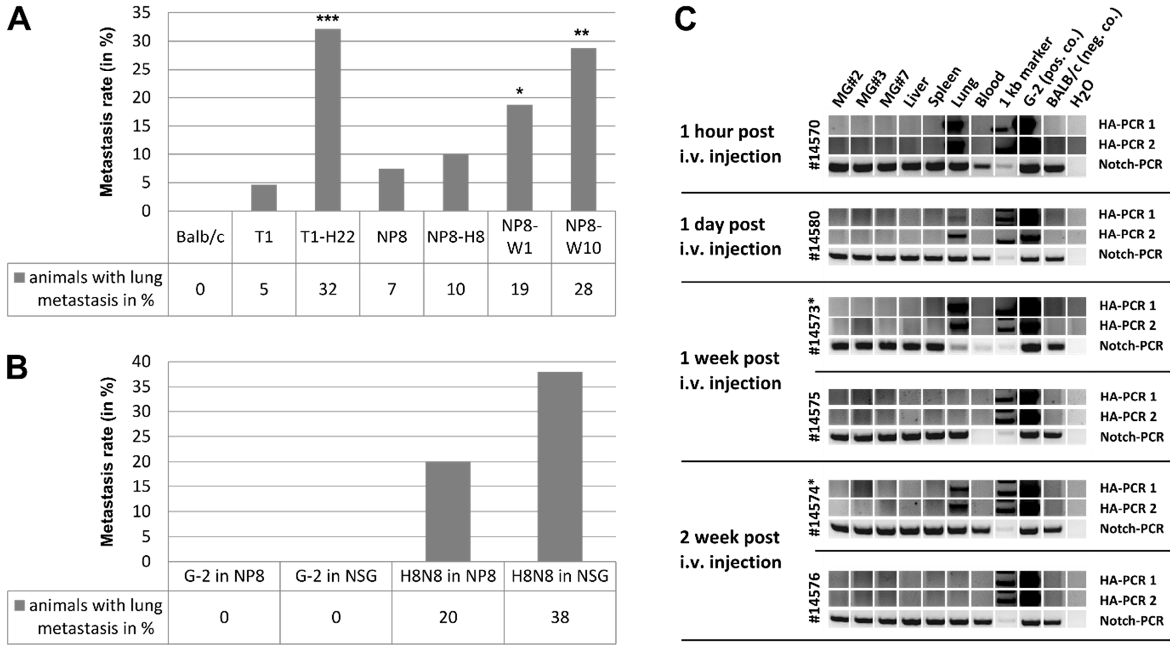

Figure S1: Metastasis of primary WAP-T tumors and transplanted tumors of G-2 and H8N8 cells.

(A) Percentage of animals with lung metastasis at sacrifice (tumor size 2 cm^3) in females of the parental mouse line Balb/c (n=39), the monotransgenic mouse lines T1 (n=86) and NP8 (n=175) and the bi-transgenic mouse lines T1-H22 (n=28), NP8-H8 (n=40), NP8-W1 (n=32) and NP8-W10 (n=60) in which primary tumor growth was induced by mating. Fischer's exact test: T1 vs T1-H22: p = 0.0004; NP8 vs NP8-W1: p = 0,040; NP8 vs NP8-W10: p = 0,002. Data described in [15] **(B)** Percentage of animals with lung metastasis at sacrifice (tumor size 2 cm^3) in female NP8 and Nod/Scid-gamma mice orthotopically transplanted with G-2 (n=29 and 14 respectively) or H8N8 cells (n= 20 and 13 respectively) into mammary gland #3; **(C)** Exemplary results of PCRs in mouse tissues prepared at the designated time points after TV injection of 10^5 G-2 cells. HA-PCR for detection of DTC, Notch PCR signal was used as control for DNA input. * animals with confirmed metastasis in immune histology.
