# Supplementary figures and images for: Epithelial-mesenchymal plasticity is a decisive feature for the metastatic outgrowth of disseminated WAP-T mouse mammary carcinoma cells
Source: BMC Cancer. 2015 Mar 26;15:178. doi: 10.1186/s12885-015-1165-5 (PMC4381675; doi:10.1186/s12885-015-1165-5)

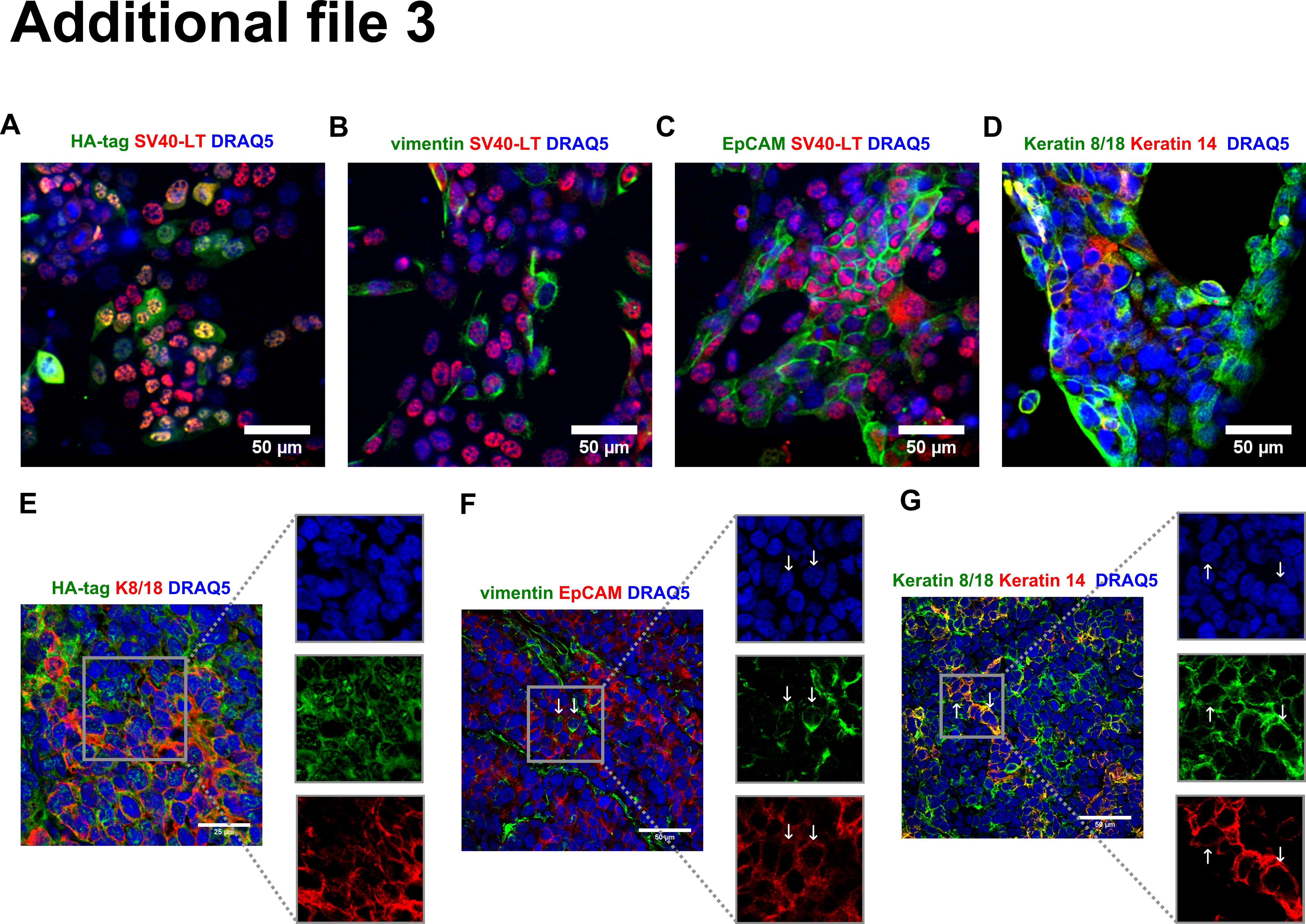

Supplement: Additional file 3: Figure S2. — Immunofluorescence characterization of H8N8 cells. [file 12885_2015_1165_MOESM3_ESM.png]
